# Mapping Potential Risks for the Transmission of Spotted Fever Rickettsiosis after Environmental Changes in an Atlantic Forest Region in Brazil

**DOI:** 10.1021/acsomega.6c03352

**Published:** 2026-04-29

**Authors:** Ana Luiza Fonseca Destro, Joice de Melo Agripino, Lara Maria Barbosa Marquezini, Ana Íris de Lima Duré, Talita Émile Ribeiro Adelino, Karla Bitencourth, Christiane Mariotini-Moura, Michelle Dias de Oliveira Teixeira, Juliana Lopes Rangel Fietto, Cláudio Lísias Mafra de Siqueira, Eduardo Lázaro de Faria da Silva, Igor Cunha Lima Acosta, Yhuri Cardoso Nóbrega, Marcelo Renan de Deus Santos, Raphael de Souza Vasconcellos

**Affiliations:** † Federal University of Viçosa (UFV), Department of Biochemistry and Molecular Biology, Viçosa 36570-900, MG, Brazil; ‡ Federal University of Viçosa (UFV), Department of Medicine and Nursing, Viçosa 36570-900, MG, Brazil; § University of São Paulo (USP), Department of Preventive Veterinary Medicine and Animal Science, São Paulo 05403-000, SP, Brazil; ∥ Marcos Daniel Institute (IMD), Serra 29161-280, Espírito Santo, Brazil; ⊥ Ezequiel Dias Foundation (FUNED), Belo Horizonte 30510-010, Brazil; # Laboratory of Ticks and Other Wingless Arthropods (LAC), Oswaldo Cruz Institute, 18 Oswaldo Cruz Foundation (FIOCRUZ)-RJ, Rio de Janeiro 21040-900, Brazil

## Abstract

Brazilian Spotted Fever (BSF), caused by *Rickettsia rickettsii*, is a fatal tick-borne disease endemic to southeastern Brazil. This study evaluates the eco-epidemiology of BSF in Santa Cruz do Escalvado, Minas Gerais, a region impacted by significant environmental changes following the 2015 Fundão dam collapse. We conducted serological and molecular surveys of mammals (*n* = 97) and ticks (*n* = 1,123) to assess pathogen circulation. Serological analysis revealed a 27.5% prevalence of anti-*R. rickettsii* antibodies in dogs, a marked increase compared to historical data (2% in 2009) from the same region. Conversely, wildlife sentinels (opossums, capybaras) showed minimal seroreactivity. Molecular screening of ticks identified *Rickettsia bellii* and a *Rickettsia tamurae*/*Rickettsia monacensis*-like genotype in *Amblyomma dubitatum* and *Amblyomma sculptum*, respectively; however, *R. rickettsii* DNA was not detected in any vector. These findings suggest a dichotomy in transmission cycles: a peri-urban cycle with high canine exposure driven by *A. sculptum*, and a riparian cycle involving *A. dubitatum* harboring non-*R. rickettsii* agents. The discrepancy between high canine seropositivity and the absence of *R. rickettsii* in vectors suggests that cross-reactivity with emerging SFG rickettsiae may be confounding surveillance signals, highlighting the need for specific molecular diagnostics in risk assessment.

## Graphical abstract

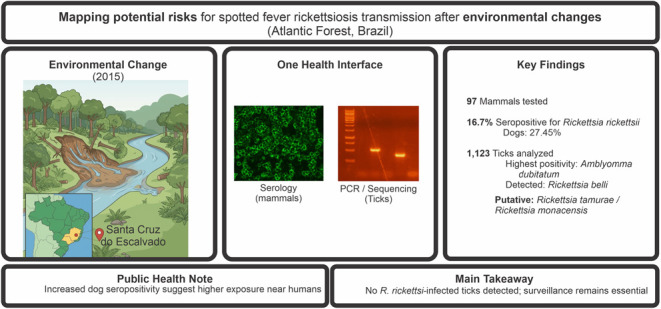

## 1 Introduction

Rickettsiae are small, Gram-negative, obligate intracellular bacteria (order Rickettsiales, family Rickettsiaceae) measuring approximately 0.3–0.5 μm in width and 0.8–2.0 μm in length. These organisms are maintained in enzootic cycles involving mammals and hematophagous arthropods such as ticks, mites, fleas, and lice.^1^ Over evolutionary time, *Rickettsia* species have developed diverse adaptations enabling survival in varied environments, employing multiple arthropod vectors and mammalian hosts, with interactions ranging from mutualistic to pathogenic.^1^

Rickettsiae are classically grouped according to genomic relatedness, antigenic properties, and clinical characteristics into the spotted fever group (SFG), typhus group (TG), transitional group (TRG), and ancestral group (AG).^1–3^ The SFG includes more than 30 recognized species, such as *Rickettsia rickettsii*, the agent of Rocky Mountain spotted fever (RMSF);^4^ *Rickettsia conorii*, which causes Mediterranean spotted fever;^5^ *Rickettsia africae*, associated with African tick-bite fever; and *Rickettsia australis*, the cause of Queensland tick typhus^6–8^

Brazilian spotted fever (BSF), caused by *R. rickettsii*, remains one of the most severe tick-borne bacterial infections worldwide because of its high case-fatality rate in endemic settings. Nationwide surveillance in Brazil (2007–2015) recorded 1,245 confirmed cases, with an overall case-fatality rate (CFR) of approximately 33%, reaching 55% in the Southeast region.^9^ In São Paulo, a referral-center series documented a 30% CFR (lower than statewide figures and attributed to heightened diagnostic suspicion and timely therapy), whereas broader state data have at times shown substantially higher lethality.^10,11^ In Minas Gerais, CFR was ∼40% during 2000–2008, and more recent analyses (2011 onward) still indicate high lethality (∼33%) alongside increasing case counts^12,13^ This pattern aligns with the recognized high pathogenicity of *R. rickettsii* in the SFG and with international alerts highlighting marked mortality when diagnosis and doxycycline treatment are delayed.^9^

From 2007 to 2024, 459 laboratory-confirmed BSF cases were reported in Minas Gerais, a state encompassing both the Cerrado and Atlantic Forest biomes.^14^ In this region, *Amblyomma sculptum* is the most prevalent human-biting tick and the main vector of *R. rickettsii*.^15,16^ Although *A. sculptum* is typically absent from dense Atlantic Forest tracts,^17–19^ it has become established in degraded forest fragments.^17,20^ In such settings, forest degradation and mosaics of open and wooded habitats likely create Cerrado-like microclimates that favor tick survival.^21,22^ Historically, within Minas Gerais, BSF has been more frequent in Atlantic Forest regions, including the Zona da Mata and the valleys of the Doce, Jequitinhonha, and Mucuri rivers, as well as the central region around Belo Horizonte, an ecotone between the Atlantic Forest and Cerrado.^23,24^

Santa Cruz do Escalvado is a municipality in the Piranga Valley with a documented BSF history. It covers 258.726 km^2^ and a population of 4673 people (2022 Census), approximately 80% of whom live in rural settings and are primarily engaged in agriculture.^25^ Despite reported BSF cases, surveillance classified it as a “silent focus” from 2004 to 2018, a period that nonetheless included two confirmed fatal cases.^26^

In 2004, the municipality underwent substantial landscape modification due to the construction of the Risoleta Neves hydroelectric power plant in Doce River.^27^ In 2015, the Fundão tailings dam (Bento Rodrigues, Mariana, Minas Gerais) collapsed, releasing ∼40 million m^3^ of iron-ore tailings into the Doce River basin, with extensive vegetation loss, housing destruction, and severe ecological impacts. Roughly 115 km downstream, the Risoleta Neves Hydroelectric Plant (also known as Candonga), retained ∼10.5 million m^3^ of tailings. Subsequently, the reservoir level was lowered urgently to manage the sediment wave.^28^

Recent environmental change has facilitated the expansion and colonization of ticks into new geographic areas that have become important hotspots for tick-borne diseases (TBDs).^1,29^ In Brazil, large infrastructure projects have been linked to the emergence of endemic diseases, including malaria, yellow fever, dengue, leishmaniases, and other arboviral infections.^30^ Such events often reflect the displacement or increased proximity of vertebrate and invertebrate hosts previously confined to natural habitats, thereby intensifying contact with susceptible hosts and expanding the geographic spread of pathogens. This process includes increases in rickettsioses and borrelioses.^31,32^

Against this backdrop, we conducted an eco-epidemiological assessment in Santa Cruz do Escalvado, Minas Gerais, Brazil, to determine whether recent ecological transformation in the area has altered the potential risk of BSF transmission.

## 2 Materials and Methods

### 2.1 Ethics Statement

All procedures were approved by the Brazilian Biodiversity Authorization and Information System (SISBIO no. 88045–1) and by the Institutional Animal Care and Use Committee of the Federal University of Viçosa (CEUA-UFV, protocol 29/2023).

### 2.2 Study Area

Santa Cruz do Escalvado (20°14′00.0″S, 42°49′00.0″W; elevation 419 m) is located in the Doce River valley (Zona da Mata, Minas Gerais, Brazil), within the Atlantic Forest biome. The climate is humid subtropical, with mean temperatures of approximately 23 °C during the rainy season (October–February) and 19 °C in the dry season (March–September). Four localities were selected based on the following criteria: (i) history of BSF cases, (ii) presence of water bodies and capybaras and (iii) a gradient of anthropization from urban to rural environments: Nova Soberbo, Porto Plácido, Santa Cruz (urban and peri-urban sectors), and Viana. At these sites, the presence of amplifier hosts and tick vectors was confirmed through direct observation and field surveys.

Sampling points encompassed riparian zones along the Doce River, rural properties characterized by monoculture plantations, backyards containing secondary vegetation, fishing areas, soccer fields and other sites frequently visited by domestic and wild hosts. This sampling strategy allowed for targeted collection in zones with higher BSF transmission potential, while ensuring representativeness of the collected data across different landscape types.

### 2.3 Historical Survey of Human Cases

Epidemiological data covering the period between 2010 and 2024 were obtained from Brazil’s Notifiable Diseases Information System (SINAN) and cross-validated by the Ezequiel Dias Foundation (FUNED) for the Regional Health Administration of Ponte Nova.

### 2.4 Sampling Design

Minimum sample sizes were calculated using EpiTools (https://epitools.ausvet.com.au/) with a 90% confidence level and 10% precision, assuming an expected seroprevalence of 20% in dogs and 5% in equids. The resulting targets were ≥44 dogs and ≥13 equids, of which 51 and 23 were actually sampled, respectively. Additionally, according to the Brazilian Ministry of Health Technical Note No. 41/2023-CGZV/DEDT/SVSA/MS, in areas infested with the ticks *A. sculptum*, *Amblyomma aureolatum*, *Amblyomma ovale*, or *Amblyomma tigrinum*, sentinel sampling should be conducted following host-specific parameters: horses— 15 animals; dogs—30 animals; and capybaras—sampled in a number representative of band size, using the formula $n=\frac{83\times N}{83+N}$ , where n is the sample size and N is the number of adult individuals.^33^ Three field campaigns, each lasting 15 days, were conducted bimonthly in January, May, and September 2024.

### 2.5 Vertebrate Trapping and Sampling

For small to medium mammals, three Tomahawk and three Sherman traps per capture station were deployed at least 10 m apart, totaling 65 active traps per day during each campaign. Traps operated continuously for 13 consecutive days with daily inspection rather than round-the-clock checks. Baits (banana, peanut butter, sardines, and cornmeal) were replenished daily. Captured animals were identified, tagged with numbered ear labels, measured for biometric parameters and sampled for blood and ticks, before being released at the capture site.

For capybaras (*Hydrochoerus hydrochaeris*), three custom—built metal traps equipped with pedal-activated guillotine doors were installed. Animals were attracted with fruit and sugar cane offered for 30 days prior to capture attempts. Chemical immobilization consisted of ketamine (6 mg/kg), midazolam (1 mg/kg), and tramadol (8 mg/kg), with continuous monitoring of heart rate, respiratory rate, and temperature. After full anesthetic recovery, animals were released at the capture site.

Dog and equid sampling were performed in urban and rural zones. Venipuncture sites varied according to species and body size: opossums (tail vein), capybaras (cephalic vein), dogs and equids (jugular or cephalic vein). Blood samples were collected into gel-separator tubes and maintained under refrigeration until centrifugation. Serum samples were aliquoted into microtubes and stored at −20 °C for subsequent analyses at the Laboratory of Parasitology, Epidemiology, and Virology (LAPEV), Federal University of Viçosa (UFV), Viçosa, MG. Noncanine serum samples intended for indirect immunofluorescence assay (IFA) were transported frozen to the Zoonoses and Vector-Borne Diseases Laboratory (São Paulo City Health Department) under UFV supervision. Canine samples were analyzed at LAPEV/UFV.

### 2.6 Tick Collection

Tick collection was performed using a combined approach, which included: white-cloth dragging (800 m per sampled area), dry ice (CO_2_) traps to attract free living ticks, systematic visual inspection of the vegetation and ground substrates and direct examination of captured hosts. Sampling was performed in all four seasons. Larvae were pooled in groups of ten, whereas nymphs and adults were stored individually in 70% ethanol.

### 2.7 Serology (Indirect Immunofluorescence Assay, IFA)

Mammalian serum samples were tested individually by IFA following an in-house protocol adapted from Pacheco.^34^ Briefly, serum samples were incubated for 30 min at 37 °C in a humid chamber on slides previously fixed with *R. rickettsii* antigen. Slides were subsequently washed in phosphate-buffered saline (PBS) for 5 min to remove unbound material and reincubated under the same conditions with FITC-conjugated species-specific anti-IgM secondary antibody diluted in PBS containing Evans blue (0.01 mg/mL) to allow specific visualization of serum antibodies.

The protocol was standardized in-house using human serum samples positive for BSF provided by the FUNED, ensuring reagent reliability and assay performance consistency. All tests were performed in technical duplicate, with one serum sample available per animal. Assay controls included PBS (blank negative control), reactive serum (positive control), and nonreactive serum (negative control). All samples were initially screened at a dilution 1:64 and reactive samples subjected to 2-fold dilutions to determine end point titers.

Slides were examined under an EVOS fluorescence microscope (Life Technologies) equipped with a GFP filter set to detect anti-*R. rickettsii* antibodies. To ensure representative sampling, five fluorescence images per well were acquired (four peripheral fields and one central field).

### 2.8 Tick Identification

Ticks collected from hosts and from the environment were morphologically identified to the species level under a stereomicroscope (Nikon SMZ800), using dichotomous keys for adult Ixodidae^35^ and *Amblyomma* nymphs. Due to limitations in the available taxonomic references, *Amblyomma* larvae were identified only to the genus level.^36^ After identification, adult and nymphal ticks were counted individually by species, whereas larvae were grouped into pools of ten individuals.

### 2.9 DNA Extraction

Genomic DNA was extracted from tick samples. Specimens were washed three times in sterile distilled water and subsequently incubated at 56 °C for approximately 15 min. Ticks were then dissected with disposable scalpel blades and mechanically macerated to enhance extraction efficiency. DNA extractions were carried out using the PureLink Viral RNA/DNA Mini Kit (Invitrogen, catalog no.12280–050). Extracted DNA was stored at −20 °C until molecular analyses. Adults and nymphs were processed individually, whereas larval pools comprised ten specimens each.

### 2.10 Molecular Screening

To validate the DNA extraction process, a conventional PCR targeting the tick 16S rRNA gene was performed and samples that showed successful amplification were subsequently used in downstream assays.

Real-time PCR (qPCR) was performed using primers CS-5 (forward), CS-6 (reverse) and probe CS (Table 1) to amplify a fragment of the *gltA* (citrate synthase) gene of *Rickettsia* spp. Reactions were carried out on a StepOne Real-Time PCR System (Applied Biosystems) in a total volume of 15 μL containing 8.25 μL GoTaq Probe 1-Step Master Mix, 0.74 μL of each primer (10 μM), 0.74 μL of probe (15 μM) and 2.72 μL DNase-free water. Nuclease-free water and DNA extracted from *R. rickettsii*-infected VERO cells served as negative and positive controls, respectively.

**Table 1:** Primer Sequences Used in the Molecular Analyses

primer	sequence	references
Cs5–Fw	GAGAGAAAATTATATCCAAATGTTGAT	Labruna et al.^41^
Cs6–Rv	AGGGTCTTCGTGCATTTCTT	Labruna et al.^41^
GltA–Fw	GCAAGTATCGGTGAGGATGTAAT	Labruna et al.^41^
GltA–Rv	GCTTCCTTAAAATTCAATAAATCAGGAT	Labruna et al.^41^
OmpA–Fw	ATGGCGAATATTTCTCCAAAA	Regnery e Plikaytis, 1991
OmpA–Rv	AGTGCAGCATTCGCTCCCCCT	Regnery e Plikaytis, 1991
16S–Fw	CCGGTCTGAACTCAGATCAAGT	Mangold et al. 1998
16S–Rv	GCTCAATGATTTTTTAAATTGCTGT	Mangold et al. 1998

Samples testing positive by qPCR were subsequently subjected to conventional PCR targeting the *gltA* and *ompA* genes (the latter specific to spotted fever group *Rickettsia*). Ten-microliter reactions were performed in a Biocycler MJ-25 thermocycler, containing 1 μL of template DNA, 5 μL GoTaq Green Master Mix (M712), 1 μL of each primer (10 μM) and 2 μL DNase-free water. Cycling conditions were as follows: 95 °C for 3 min; 35 amplification cycles of 95 °C for 30 s, 60 °C for 30 s, and 72 °C for 30 s; followed by a final extension at 72 °C for 5 min. Each run included the same negative (nuclease-free water) and positive (*R. rickettsii* VERO-cell DNA) controls as used in the qPCR. Primer sequences are listed in Table 1. Amplicons were resolved by electrophoresis on 1.5% agarose gels stained with ethidium bromide (0.1 μg/mL) and visualized under UV transillumination.

### 2.11 Nanopore Sequencing

Total genomic DNA from the 16 positive samples was quantified and used for library preparation with the Ligation Sequencing Kit (Oxford Nanopore Technologies, ONT) and Native Barcoding Kit (ONT), followed by sequencing on the MinION platform (ONT) using an R9.4 flow cell. Raw FAST5 files were processed using Guppy v6.0 (ONT) for basecalling and demultiplexing. Reads were assembled into gene-specific consensus using reference fragments deposited in the National Center for Biotechnology Information (NCBI) database (*gltA*: NZ_CP040325.1; 17-kDa antigen: MK509751.1; *ompA*: MK102719.1) and processed through a custom bioinformatic pipeline integrating the following tools: NanoPlot (v1.43.0; github.com/wdecoster/NanoPlot) for read-quality assessment; Chopper (v0.8.0) for adapter and low-quality trimming; Minimap2 (v2.28) for read alignment; Samtools (v1.20) for alignment handling; Bcftools (v1.20) for variant calling; iVar (v1.4.2) for consensus generation and Pilon (v1.24) for polishing and refinement. Following assembly, consensus sequences were taxonomically assigned to the genus or species level based on sequence similarity using NCBI BLASTn. To corroborate BLAST-based identifications and explore evolutionary relationships, phylogenetic analyses were performed using a concatenated data set of *gltA* + *htr*A + *ompA* comprising newly generated sequences and representative references sequences of multiple *Rickettsia* species retrieved from NCBI. Maximum-likelihood phylogenetic reconstruction was carried out in PhyML v3.0 (Guindon et al., 2010) under the T92+G substitution model and branch support was assessed using the approximate likelihood ratio test (aLRT) with 1,000 replicates (Anisimova and Gascuel, 2006).

## 3 Results

### 3.1 Demographic and Spatial Characterization and Environmental Disturbances in the Region

Santa Cruz do Escalvado, located in Minas Gerais state, has a population of 4673 inhabitants distributed over an area of 258.726 km^2^, resulting in a population density of 18.06 inhabitants per km^2^. This region has been affected by major disturbances since 2015, including hydrological changes associated with the Risoleta Neves reservoir and the downstream dispersal of tailings from the Fundão dam collapse along the Doce River (Figure 1). These factors may influence local vector–host dynamics, but this remains a working hypothesis because baseline data prior to the disturbance are not available for robust comparison.

**Figure 1:**
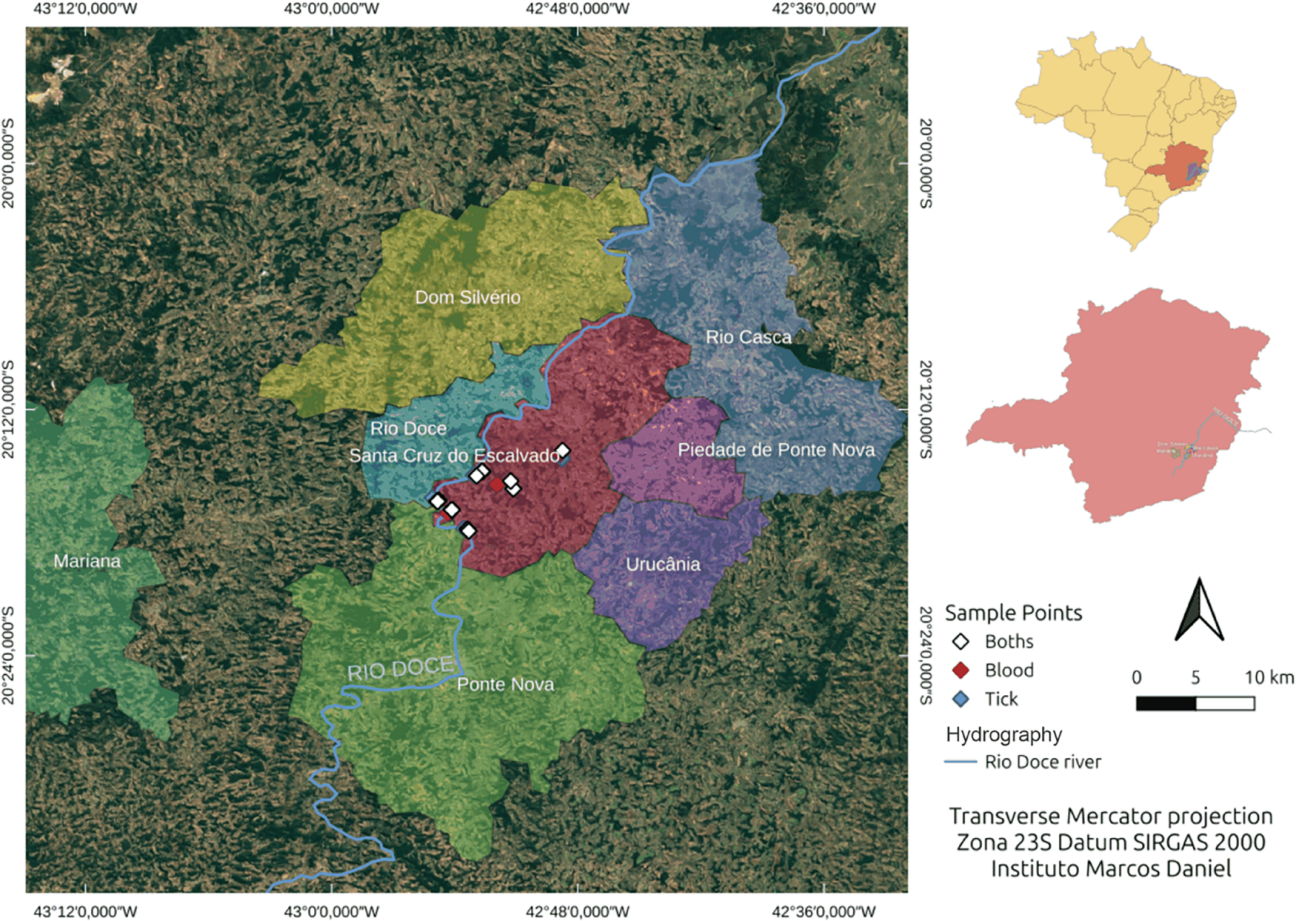
Study area in the Doce River basin, highlighting Santa Cruz do Escalvado (red) and neighboring municipalities (Rio Doce, Ponte Nova, Dom Silvério, Rio Casca, Piedade de Ponte Nova, and Urucânia), the course of the Doce River, and the georeferenced sampling sites according to sample type (blood, ticks, or both). Figure 1 was kindly prepared by Victor Vale.

### 3.2 Selection of Localities and Regional Case Series

This study encompassed four areas within the municipality of Santa Cruz do Escalvado: Nova Soberbo, Porto Plácido, the urban and peri-urban zones of Santa Cruz do Escalvado, and Viana, selected based on the local history of BSF cases, the presence of water bodies and capybaras population, and their representation along an urban–rural anthropization gradient, to capture variation in land use. Sampling points included riparian margins along the Doce River, rural properties, backyards with secondary vegetation, fishing sites and soccer fields, settings where field surveys confirmed the circulation of both domestic and wild hosts (Figure 1).

Santa Cruz do Escalvado falls under the jurisdiction of the Regional Health Administration of Ponte Nova, which forward suspected BSF samples to the FUNED for laboratory analysis and confirmation. According to FUNED’s case records, this jurisdiction reported laboratory confirmed cases in 2011 (*n* = 2), 2015 (*n* = 1), 2017 (*n* = 3), 2018 (*n* = 6), 2019 (*n* = 3), and 2023 (*n* = 3). Of these, only one case (in 2018) occurred in Santa Cruz do Escalvado, and resulted in a fatal.

### 3.3 Seroprevalence in Domestic and Wild Mammals

A total of 22 opossums (*Didelphis aurita*), 51 domestic dogs (*Canis lupus familiariz*), and 23 equids (17 horses, 5 mules, and 1 donkey) were sampled across all study sites, in addition to 1 capybara (*H. hydrochaeris*) captured during the dry season in Porto Plácido (Table 2). The seroprevalence of *R. rickettsii* antibodies in dogs was significantly higher than in all other sampled mammals species (Figure 2 and Table 2). Compared with combined noncanine mammals, dogs showed higher seroreactivity, probably associated with their peridomestic habits and frequent contact with ticks. This finding reinforces the role of dogs as epidemiological sentinels and highlights the importance of implementing targeted surveillance and control.

**Figure 2:**
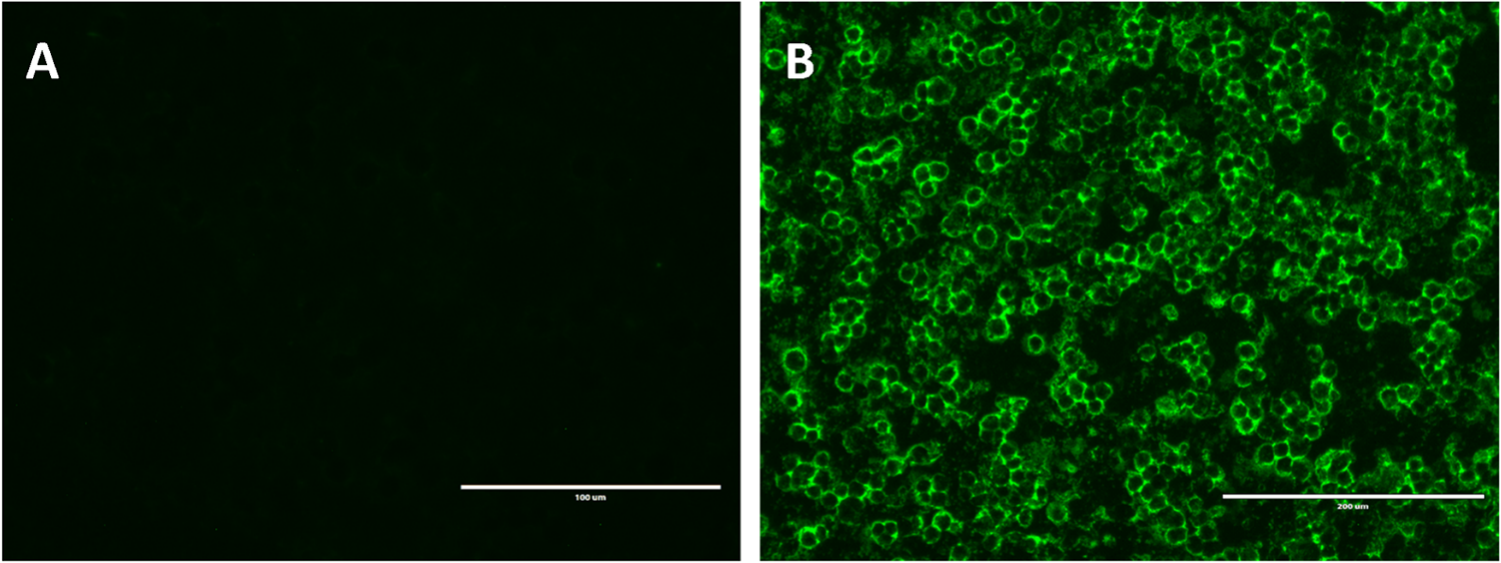
Indirect immunofluorescence assay (IFA) for dog sera. (A) Negative control. (B) Reactive sample (1:256). IFA was performed on all animal samples collected; the figure illustrates a representative assay.

**Table 2:** Indirect Immunofluorescence Assay (IFA) Results for Anti-*R. rickettsii* Antibodies in Animal

species	tested	positive	%	location of positive cases	antibody titers (no. of animals)
*Canis lupus familiariz*	51	2 12	3.9 23.5	Porto Plácido Santa Cruz (urban)	256(2) 64(8); 128(3); 256(1)
*D. aurita*	22	1	4.5	Santa Cruz (urban)	64(1)
*Equus asinus*	01	0	0		
*Equus caballus*	17	1	5.9	Viana	64(1)
Hybrid of *Equus caballus Equus asinus*	05	0	0		
*H. hydrochaeris*	01	0	0		
Total	**97**	**16**	**16.7**		

### 3.4 Tick Community and Seasonality

Overall tick activity peaked in autumn and winter, with substantially lower counts in spring and summer (Figure 3A). At the species level, *A. sculptum* predominated across all seasons, reaching its highest abundance in autumn, remaining high in winter and summer, and showing a marked decline in spring (Figure 3B). Specimens classified as *Amblyomma* sp. were concentrated in autumn and winter, absent in spring, and rare in summer (Figure 3B). *Rhipicephalus sanguineus* sensu lato was not detected in winter and increased steadily from autumn (4 specimens) to spring (14) and summer (36). *Rhipicephalus microplus* was recorded only in autumn (26). *A. dubitatum* was most frequent in summer (25), with sporadic detections in other seasons. Overall, these findings underscore the persistent dominance of *A. sculptum* and a pronounced seasonal pattern, with tick abundance peaking in autumn and winter.

**Figure 3:**
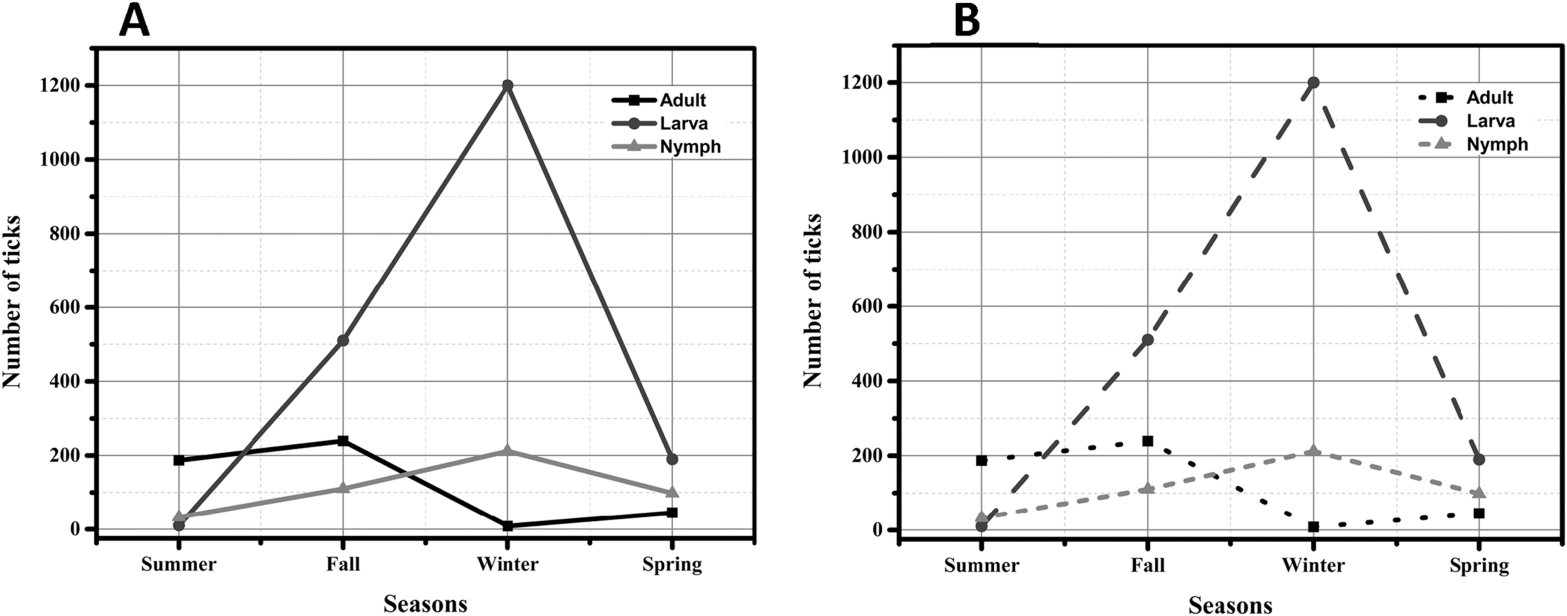
Seasonal dynamics of ticks in Santa Cruz do Escalvado (Minas Gerais, Brazil). (A) Number of ticks collected by season, stratified by life stage (adult, larva, nymph). (B) Seasonal distribution by species.

### 3.5 Molecular Detection of *Rickettsia* spp. in Ticks

A total of 1,123 ticks were collected and screened, including 32 *A. dubitatum*, 823 *A. sculptum*, 188 pools of *Amblyomma* spp., 26 *R. microplus*, and 54 *R. sanguineus* sensu lato. All ticks collected from opossums, equids, and dogs were negative for *Rickettsia* DNA. Among the 19 ticks collected from a capybara at Porto Plácido, one female and two male *A. dubitatum* were positive for *Rickettsia* spp. during the dry season. From vegetation, 13 *A. dubitatum* individuals were positive in Nova Soberbo and Porto Plácido. In addition, two *A. sculptum* nymphs from Nova Soberbo were positive during the rainy season. All other assays were negative (Table 3).

**Table 3:** PCR Results for Detection of *Rickettsia* spp. in Ticks, Stratified by Season, Life Stage, Genus, and Species, from Porto Plácido and Nova Soberbo, Minas Gerais, Brazil (2024)

			marker				
location	period	tick	gltA	Cs5	ompA	genus/species	vegetation/host
Novo Soberbo	Rainy	*A. dubitatum* (Female)	Negative	Positive	Negative	*Rickettsia*	Vegetation
Novo Soberbo	Dry	*A. dubitatum* (Female)	Positive	Positive	Negative	*Rickettsia bellii*	Vegetation
Novo Soberbo	Rainy	*A. dubitatum* (Male)	Positive	Positive	Negative	*R. bellii*	Vegetation
Novo Soberbo	Rainy	*A. dubitatum* (Male)	Negative	Positive	Negative	*R. bellii*	Vegetation
Novo Soberbo	Rainy	*A. dubitatum* (Male)	Negative	Positive	Negative	*Rickettsia*	Vegetation
Novo Soberbo	Rainy	*A. dubitatum* (Male)	Negative	Positive	Negative	*Rickettsia*	Vegetation
Novo Soberbo	Rainy	*A. dubitatum* (Male)	Negative	Positive	Negative	*Rickettsia*	Vegetation
Novo Soberbo	Rainy	*A. dubitatum* (Male)	Positive	Positive	Negative	*R. bellii*	Vegetation
Novo Soberbo	Rainy	*A. dubitatum* (Nymph)	Negative	Positive	Negative	*Rickettsia*	Vegetation
Novo Soberbo	Rainy	*A. dubitatum* (Nymph)	Negative	Positive	Negative	*Rickettsia*	Vegetation
Novo Soberbo	Rainy	*A. sculptum* (Nymph)	Positive	Positive	Negative	*Rickettsia*	Vegetation
Novo Soberbo	Rainy	*A. sculptum* (Nymph)	Negative	Positive	Negative	*Rickettsia*	Vegetation
Porto Plácido	Dry	*A. dubitatum* (Female)	Positive	Positive	Negative	*Rickettsia*	Capybara
Porto Plácido	Dry	*A. dubitatum* (Female)	Positive	Positive	Negative	*Rickettsia*	Capybara
Porto Plácido	Dry	*A. dubitatum* (Male)	Positive	Positive	Negative	*Rickettsia*	Capybara
Porto Plácido	Dry	*A. dubitatum* (Male)	Positive	Positive	Negative	*Rickettsia*	Capybara

Of the 16 positive samples subjected to sequencing, five (samples 45, 46, 49, 249, and 1245) showed >99.00% identity with *R. bellii*. In the phylogenetic reconstruction, these sequences clustered with *R. bellii* and all originated from *A. dubitatum* collected in Nova Soberbo. The remaining sequences showed 99.54–100% identity with *Rickettsia* sp. and grouped within a clade whose sister taxa were *Rickettsia tamurae* and *Rickettsia monacensis* (Figure 4 and Supporting Information).

**Figure 4:**
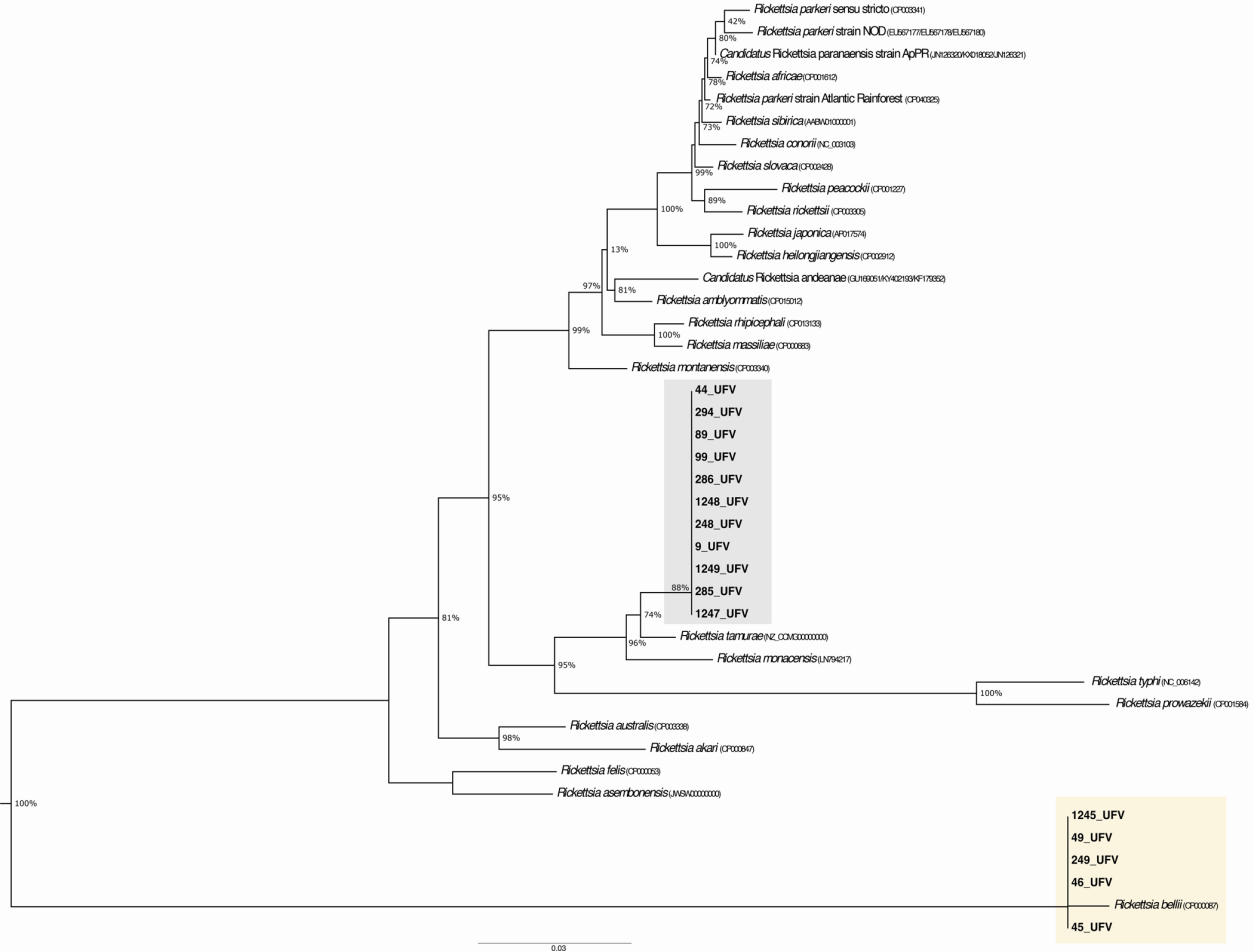
Concatenated phylogeny, inferred by maximum likelihood analysis with evolution model T92 + G, of rickettsia *glt*A, *htr*A and *omp*A genes fragmente (773+ 434+ 635 bp) detected in ticks collected from Porto Plácido and Nova Soberbo, Minas Gerais, Brazil. Gray and yellow shaded areas mark the clades containing the sequences generated in this study at Fundação Ezequiel Dias (FUNED). The numbers on the branches represent support values (aLRT, 70% cutoff). The scale bar denotes substitutions per site.

## 4 Discussion

This study provides the first comprehensive and contemporary eco-epidemiological assessment of *Rickettsia* circulation in Santa Cruz do Escalvado after the Doce River environmental disaster. Historical data from 1994 reported seropositivity to *R. rickettsii* in 7.14% of humans and 13.8% of dogs, confirming pathogen circulation in the region.^37^ Case surveillance records indicated an increase in spotted fever notifications after the Fundão dam collapse; however, this rise may not be directly attributable to the disaster itself, as heightened awareness and improved diagnostic capacity likely contributed to the apparent increase. In the indirect IFA, only one opossum and one horse tested positive for *R. rickettsii*. Both species are considered informative sentinel hosts, as they frequently sustain tick infestations.^38^ Horses, in particular, often live in close proximity to humans and can support heavy tick burdens thereby increasing the potential for human exposure, similar to dogs.^38^ Nevertheless, the small number of positive horses and opossums, combined with low antibody titers, aligns with the absence of *R. rickettsii* DNA in the sampled ticks. A 2010 IFA survey in opossums from Santa Cruz do Escalvado did not detect *R. rickettsii* or *Rickettsia bellii*, but identified *R. parkeri*, *R. amblyommii*, and *Rickettsia felis*. The same study detected *R. felis* by DNA analyses, with 8 positives among 646 samples.^38^ In contrast, our analyses did not detect *R. felis*. Another study (2009) in the same area reported 14.3% positivity for *R. rickettsii* and *R. parkeri* in opossums, 5.4% for *R. bellii*, and 2.7% for SFG rickettsiae in horses.^39^ Unlike our findings, the lowest seropositivity in that study occurred in dogs (2% for *R. rickettsii* and 2% for *R. parkeri*) in 2009,^39^ whereas our canine serum samples reached 27.5% seropositivity for *R. rickettsii*. This marked increase is of public health concern given the close contact between dogs and humans in household environments. Dogs therefore remain key sentinels for early detection of BSF risk. Moreover, the low IFA reactivity in equids and opossums in our study, together with the absence of positivity in capybara, may reflect spatial/temporal heterogeneity of exposure or waning titers after previous transmission waves, a plausible scenario in settings with focal and seasonal transmission.

The apparent discrepancy between canine seropositivity and the absence of *R. rickettsii* detection in ticks may be explained by several factors. First, IFA using *R. rickettsii* antigen can cross-react with antigenically related SFG species, especially when exposure involves non-*rickettsii* rickettsiae. Second, our sampling may have missed microgeographic foci where infected ticks occur at low frequency, a well-documented phenomenon reported in BSF—endemic areas.^40^ Third, temporal mismatch between serological sampling, which reflects exposure over weeks to months, and tick collection, a seasonal snapshot, may also account for the observed divergence.

Molecular and phylogenetic analyses (Figure 4, Table 3 and Supporting Information) corroborate this interpretation. In the infected ticks (16/1,123), it was detected the presence of *R. bellii* in five samples of *A. dubitatum* and *R. tamurae*/*R. monacensis*, in specimens of *A. dubitatum* and *A. sculptum*, with no detection of *R. rickettsii*. Belonging to SFG, *R. bellii* is considered nonpathogenic to humans and environmentally widespread, persisting in multiple *Amblyomma* species.^41,42^ The clustering of the remaining sequences with *R. tamurae*/*R. monacensis*, (aLRT = 96), suggests local circulation of non-*rickettsii* SFG rickettsiae, consistent with canine seropositivity via cross-reaction and with historical detections of *R. parkeri*, *R. amblyommatis*, and *R. felis* in mammals/ectoparasites from the region.^38^ In Japan, *R. tamurae* is recognized as a pathogenic etiological agent for humans, with mild symptomatology and no associated deaths.^43–45^ Although there are no confirmed cases of spotted fever associated with *R. tamurae* in South America, there is a growing detection of new rickettsia species and human rickettsioses in Brazil.^46–48^ Additionally, previous studies report the presence of rickettsias phylogenetically related to *R. tamurae* and *R. monacensis* in the state of Minas Gerais, where it was detected in a pool of free-living *Amblyomma* sp. nymphs collected in the municipality of Coronel Pacheco^48^ and in a male *A. sculptum* parasitizing a dog in the municipality of Pedro Leopoldo.^49^ There are also records of rickettsia related to *R. tamurae* and *R. monacensis* in *A. dubitatum* in the state of Rio de Janeiro,^50^ in *Ixodes fuscipes* in Paraná,^51^ in *R. microplus* in Ceará,^52^ and in *Amblyomma longirostre* larvae in Acre.^53^ Therefore, further studies are needed to understand the role of these rickettsiae related to *R. tamurae* and *R. monacensis* in Spotted Fever outbreaks in Brazil.

Among the identified ticks, *A. sculptum*. was dominant, yet molecular positivity was highest in *A. dubitatum*. *A. sculptum* is recognized as a vector of human-pathogenic SFG rickettsiae in Brazil. Moreover, *A. sculptum* poses a higher risk of biting humans; therefore, the abundance of *A. sculptum* is alarming for the potential re-establishment of disease transmission. Both *A. dubitatum* and *A. sculptum* typically feed on capybaras, which act as amplifier hosts and sentinels.,^54^ Once infected, capybaras can sustain high titers for more than three years and may pass infection to their offspring, which remain seroreactive for 1–4 months.^55^ In our study, ticks collected from capybaras were qPCR-positive for *Rickettsia* sp.; however, these strains did not show high similarity to *R. rickettsii*. Consistent with this, capybaras were not seropositive for *R. rickettsii*.

From an ecological perspective, the predominance of *A. sculptum* and the molecular detection in *A. dubitatum* delineate two partially overlapping transmission circuits. The first, of greater public-health relevance, involves *A. sculptum* in peri-urban and rural mosaics,^40^ with dogs acting as epidemiological “sensors” of landscape and management (lack of acaricide use, free roaming, herding/hunting activities).The second, primarily riparian, involves *A. dubitatum* and capybaras, maintaining non-*rickettsii* SFG rickettsiae of uncertain pathogenicity but capable of sustaining antigenic pressure in local hosts.^49–56^ The absence of seroreactivity in capybaras in this study may reflect the small sample size (*n* = 1), timing of collection, or exposure to SFG rickettsiae not captured by IFA using *R. rickettsii* antigen.

Several limitations should be acknowledged. Capybara sampling was limited, reducing inference on infection dynamic in this amplifier host. The moderate statistical support for the *R. tamurae*/*R. monacensis* clade constrains fine-scale taxonomic resolution, although the ecological interpretation remains robust. Finally, IFA with a single antigen (*R. rickettsii*) limits discrimination of exposures to other SFG rickettsiae; broader antigen panels (e.g., *R. parkeri*, *R. amblyommatis*) could reduce uncertainty due to cross-reactivity.

Because *R. bellii* is not pathogenic to humans and is less harmful than *R. rickettsii*, environmental persistence of the former is more likely than that of *R. rickettsii*.^56^ Nevertheless, Santa Cruz do Escalvado remains a risk setting, with potential for outbreaks caused by *R. rickettsii*, given the abundance of suitable hosts, the presence of competent tick vectors, and the proximity to human-occupied areas. Continuous epidemiological surveillance is therefore essential to monitor pathogen circulation and prevent severe outcomes.

## 5 Conclusions

Our findings suggest that the recent increase in Brazilian spotted fever notifications in Santa Cruz do Escalvado region likely reflects enhanced surveillance and diagnostic awareness than a true postdisaster surge in transmission. Nevertheless, the canine seropositivity to *R. rickettsii* (27.5%) indicates persistent exposure in peri-domestic settings and reinforces the role of dogs as sentinel hosts. Molecular screening of 1,123 ticks detected *R. bellii* in *A. dubitatum* and rickettsiae related to *R. tamurae*/*R. monacensis*, but no *R. rickettsii*, delineating two overlapping eco-epidemiological circuits: (i) a peri-urban/rural mosaic dominated by *A. sculptum* (higher human risk) and (ii) a riparian cycle centered on *A. dubitatum*–capybara interaction non-*rickettsii* SFG rickettsiae circulate. This landscape, characterized by abundant competent vectors, frequent human–animal contact, and ongoing environmental disturbance, remains conducive to pathogen maintenance and potential transmission.

Accordingly, continuous One Health surveillance is warranted, with priority actions including: (i) seasonal, nymph-targeted tick sampling along peri-urban interfaces and riparian corridors; (ii) sentinel serology in dogs using multiantigen IFA panels to minimize cross-reactivity; (iii) targeted vector control and habitat management around capybara movement corridors; and (iv) clinician training to ensure early recognition and prompt doxycycline treatment of suspected cases. Also, continuous community health education campaigns focusing in tick control measures and BSF prevention are essential to reduce mortality through quick response to suspected cases. Broader capybara sampling and incorporation of additional genetic markers for *Rickettsia* spp. will enhance species-level identification and refine eco-epidemiological risk assessments, supporting proactive prevention of severe disease outcomes in this ecotone.

## Supplementary Material



## Notes

Molecular identification of *Rickettsia* sequences generated from the 16 positive samples subjected to sequencing, reporting the amplified marker (gltA, htrA, and/or ompA), percent identity and alignment coverage, and the closest GenBank reference sequences with their accession numbers (PDF)
