# Supplementary material for: Mapping Potential Risks for the Transmission of Spotted Fever Rickettsiosis after Environmental Changes in an Atlantic Forest Region in Brazil
Source: ACS Omega. 2026 Apr 29;11(18):27648–57. doi: 10.1021/acsomega.6c03352 (PMC13177018; doi:10.1021/acsomega.6c03352)
Supplement: Supplementary file 1 [file ao6c03352_si_001.pdf]

Mapping potential risks for the transmission of spotted fever rickettsiosis after environmental changes in an Atlantic Forest region in Brazil

Ana Luiza Fonseca Destro[a], Joice de Melo Agripino[a], Lara Maria Barbosa Marquezini[a], Ana Íris de Lima Duré[e], Talita Émile Ribeiro Adelino[e], Karla Bitencourth[f], Christiane Mariotini-Moura[b], Michelle Dias de Oliveira Teixeira[a], Juliana Lopes Rangel Fietto[a], Cláudio Lísias Mafra de Siqueira[a], Eduardo Lázaro de Faria da Silva[a], Igor Cunha Lima Acosta[c], Yhuri Cardoso Nóbrega[d], Marcelo Renan de Deus Santos[d], Raphael de Souza Vasconcellos[a].

[a] Federal University of Viçosa (UFV), Department of Biochemistry and Molecular Biology, Viçosa, MG, Brazil.

[b] Federal University of Viçosa (UFV), Department of Medicine and Nursing, Viçosa, MG, Brazil.

[c] University of São Paulo (USP), Department of Preventive Veterinary Medicine and Animal Science, São Paulo, SP, Brazil.

[d] Marcos Daniel Institute (IMD), Serra, Espírito Santo, Brazil.

[e] Ezequiel Dias Foundation (FUNED), Brazil.

[f] Laboratory of Ticks and Other Wingless Arthropods (LAC), Oswaldo Cruz Institute, Oswaldo Cruz Foundation (FIOCRUZ)-RJ, Brazil.

Corresponding author: Raphael de Souza Vasconcellos raphael.vasconcellos@ufv.br

Supporting Information Table: Molecular identification of *Rickettsia* sequences obtained from the 16 positive tick samples subjected to sequencing. For each sample, the amplified marker(s) (gltA, htrA, and/or ompA), BLASTn percent identity, alignment coverage, and the closest GenBank reference sequence(s), with the respective accession number(s), are shown.

Sample	Marker	Molecular identification
9	*htr*A	99,54% (432/434) identity with *Rickettsia* sp. Isolate 434 (OR365307)<br>99,54% (432/434) identity with *Rickettsia* sp. Isolate RNG (OR045375)<br>99,54% (431/433) identity with *Rickettsia* sp. Clone LIC 4167A (MH158235)
	*glt*A	99,87% (769/770) identity with *Rickettsia* sp. Clone LIC 9145A (MZ675720)<br>99,87% (769/770) identity with *Rickettsia* sp. Clone LIC 9145B (MZ675721)<br>99,87% (769/770) identity with *Rickettsia* sp. Clone LIC 4167A (MH158234)
	*omp*A	99,83% (589/590) identity with *Rickettsia* sp. Isolate 464 (OR365305)
44	*htr*A	99,54% (432/434) identity with *Rickettsia* sp. Isolate 434 (OR365307)<br>99,54% (432/434) identity with *Rickettsia* sp. Isolate RNG (OR045375)<br>99,54% (431/433) identity with *Rickettsia* sp. Clone LIC 4167A (MH158235)
	*glt*A	99,87% (769/770) identity with *Rickettsia* sp. Clone LIC 9145A (MZ675720)<br>99,87% (769/770) identity with *Rickettsia* sp. Clone LIC 9145B (MZ675721)<br>99,87% (769/770) identity with *Rickettsia* sp. Clone LIC 4167A (MH158234)
45	*glt*A	99,48% (766/770) identity with *Rickettsia bellii* (CP000087)
46	*glt*A	99,48% (766/770) identity with *Rickettsia bellii* (CP000087)

Sample	Marker	Molecular identification
49	*glt*A	99,48% (766/770) identity with *Rickettsia bellii* (CP000087)
89	*htr*A	99,54% (432/434) identity with *Rickettsia* sp. Isolate 434 (OR365307)<br>99,54% (432/434) identity with *Rickettsia* sp. Isolate RNG (OR045375)<br>99,54% (431/433) identity with *Rickettsia* sp. Clone LIC 4167A (MH158235)
	*glt*A	99,87% (769/770) identity with *Rickettsia* sp. Clone LIC 9145A (MZ675720)<br>99,87% (769/770) identity with *Rickettsia* sp. Clone LIC 9145B (MZ675721)<br>99,87% (769/770) identity with *Rickettsia* sp. Clone LIC 4167A (MH158234)
99	*htr*A	99,54% (432/434) identity with *Rickettsia* sp. Isolate 434 (OR365307)<br>99,54% (432/434) identity with *Rickettsia* sp. Isolate RNG (OR045375)<br>99,54% (431/433) identity with *Rickettsia* sp. Clone LIC 4167A (MH158235)
	*glt*A	99,87% (769/770) identity with *Rickettsia* sp. Clone LIC 9145A (MZ675720)<br>99,87% (769/770) identity with *Rickettsia* sp. Clone LIC 9145B (MZ675721)<br>99,87% (769/770) identity with *Rickettsia* sp. Clone LIC 4167A (MH158234)

Sample	Marker	Molecular identification
	*omp*A	99,83% (589/590) identity with *Rickettsia* sp. Isolate 464 (OR365305)
248	*htr*A	99,54% (432/434) identity with *Rickettsia* sp. Isolate 434 (OR365307)<br>99,54% (432/434) identity with *Rickettsia* sp. Isolate RNG (OR045375)<br>99,54% (431/433) identity with *Rickettsia* sp. Clone LIC 4167A (MH158235)
	*glt*A	99,87% (769/770) identity with *Rickettsia* sp. Clone LIC 9145A (MZ675720)<br>99,87% (769/770) identity with *Rickettsia* sp. Clone LIC 9145B (MZ675721)<br>99,87% (769/770) identity with *Rickettsia* sp. Clone LIC 4167A (MH158234)
	*omp*A	99,83% (589/590) identity with *Rickettsia* sp. Isolate 464 (OR365305)
249	*glt*A	99,48% (766/770) identity with *Rickettsia bellii* (CP000087)
285	*htr*A	99,54% (432/434) identity with *Rickettsia* sp. Isolate 434 (OR365307)<br>99,54% (432/434) identity with *Rickettsia* sp. Isolate RNG (OR045375)<br>99,54% (431/433) identity with *Rickettsia* sp. Clone LIC 4167A (MH158235)
	*glt*A	99,87% (769/770) identity with *Rickettsia* sp. Clone LIC 9145A (MZ675720)<br>99,87% (769/770) identity with *Rickettsia* sp. Clone LIC 9145B (MZ675721)<br>99,87% (769/770) identity with *Rickettsia* sp. Clone LIC 4167A (MH158234)

Sample	Marker	Molecular identification
286	*htr*A	99,54% (432/434) identity with *Rickettsia* sp. Isolate 434 (OR365307)<br>99,54% (432/434) identity with *Rickettsia* sp. Isolate RNG (OR045375)<br>99,54% (431/433) identity with *Rickettsia* sp. Clone LIC 4167A (MH158235)
	*glt*A	99,87% (769/770) identity with *Rickettsia* sp. Clone LIC 9145A (MZ675720)<br>99,87% (769/770) identity with *Rickettsia* sp. Clone LIC 9145B (MZ675721)<br>99,87% (769/770) identity with *Rickettsia* sp. Clone LIC 4167A (MH158234)
294	*htr*A	99,54% (432/434) identity with *Rickettsia* sp. Isolate 434 (OR365307)<br>99,54% (432/434) identity with *Rickettsia* sp. Isolate RNG (OR045375)<br>99,54% (431/433) identity with *Rickettsia* sp. Clone LIC 4167A (MH158235)
	*glt*A	99,87% (769/770) identity with *Rickettsia* sp. Clone LIC 9145A (MZ675720)<br>99,87% (769/770) identity with *Rickettsia* sp. Clone LIC 9145B (MZ675721)<br>99,87% (769/770) identity with *Rickettsia* sp. Clone LIC 4167A (MH158234)
	*omp*A	99,83% (589/590) identity with *Rickettsia* sp. Isolate 464 (OR365305)
1245	*glt*A	99,74% (768/770) identity with *Rickettsia bellii* (CP000087)

Sample	Marker	Molecular identification
1247	*glt*A	99,87% (769/770) identity with *Rickettsia* sp. Clone LIC 9145A (MZ675720)<br>99,87% (769/770) identity with *Rickettsia* sp. Clone LIC 9145B (MZ675721)<br>99,87% (769/770) identity with *Rickettsia* sp. Clone LIC 4167A (MH158234)
1248	*htr*A	99,54% (432/434) identity with *Rickettsia* sp. Isolate 434 (OR365307)<br>99,54% (432/434) identity with *Rickettsia* sp. Isolate RNG (OR045375)<br>99,54% (431/433) identity with *Rickettsia* sp. Clone LIC 4167A (MH158235)
	*glt*A	99,87% (769/770) identity with *Rickettsia* sp. Clone LIC 9145A (MZ675720)<br>99,87% (769/770) identity with *Rickettsia* sp. Clone LIC 9145B (MZ675721)<br>99,87% (769/770) identity with *Rickettsia* sp. Clone LIC 4167A (MH158234)
	*omp*A	100,00% (590/590) identity with *Rickettsia* sp. Isolate 464 (OR365305)
1249	*htr*A	99,54% (432/434) identity with *Rickettsia* sp. Isolate 434 (OR365307)<br>99,54% (432/434) identity with *Rickettsia* sp. Isolate RNG (OR045375)<br>99,54% (431/433) identity with *Rickettsia* sp. Clone LIC 4167A (MH158235)
	*glt*A	99,87% (769/770) identity with *Rickettsia* sp. Clone LIC 9145A (MZ675720)<br>99,87% (769/770) identity with *Rickettsia* sp. Clone LIC 9145B (MZ675721)<br>99,87% (769/770) identity with *Rickettsia* sp. Clone LIC 4167A (MH158234)
	*omp*A	100,00% (590/590) identity with *Rickettsia* sp. Isolate 464 (OR365305)
